# Evolutionary view of acyl-CoA diacylglycerol acyltransferase (DGAT), a key enzyme in neutral lipid biosynthesis

**DOI:** 10.1186/1471-2148-11-263

**Published:** 2011-09-20

**Authors:** Andreia C Turchetto-Zolet, Felipe S Maraschin, Guilherme L de Morais, Alexandro Cagliari, Cláudia MB Andrade, Marcia Margis-Pinheiro, Rogerio Margis

**Affiliations:** 1 Programa de Pós-Graduação em Genética e Biologia Molecular, Departamento de Genética, Universidade Federal do Rio Grande do Sul, Brazil; 2 Centro de Biotecnologia, Universidade Federal do Rio Grande do Sul, Brazil; 3 Departamento de Biofísica, Universidade Federal do Rio Grande do Sul, Brazil

## Abstract

**Background:**

Triacylglycerides (TAGs) are a class of neutral lipids that represent the most important storage form of energy for eukaryotic cells. DGAT (acyl-CoA: diacylglycerol acyltransferase; EC 2.3.1.20) is a transmembrane enzyme that acts in the final and committed step of TAG synthesis, and it has been proposed to be the rate-limiting enzyme in plant storage lipid accumulation. In fact, two different enzymes identified in several eukaryotic species, DGAT1 and DGAT2, are the main enzymes responsible for TAG synthesis. These enzymes do not share high DNA or protein sequence similarities, and it has been suggested that they play non-redundant roles in different tissues and in some species in TAG synthesis. Despite a number of previous studies on the DGAT1 and DGAT2 genes, which have emphasized their importance as potential obesity treatment targets to increase triacylglycerol accumulation, little is known about their evolutionary timeline in eukaryotes. The goal of this study was to examine the evolutionary relationship of the DGAT1 and DGAT2 genes across eukaryotic organisms in order to infer their origin.

**Results:**

We have conducted a broad survey of fully sequenced genomes, including representatives of Amoebozoa, yeasts, fungi, algae, musses, plants, vertebrate and invertebrate species, for the presence of DGAT1 and DGAT2 gene homologs. We found that the DGAT1 and DGAT2 genes are nearly ubiquitous in eukaryotes and are readily identifiable in all the major eukaryotic groups and genomes examined. Phylogenetic analyses of the DGAT1 and DGAT2 amino acid sequences revealed evolutionary partitioning of the DGAT protein family into two major DGAT1 and DGAT2 clades. Protein secondary structure and hydrophobic-transmembrane analysis also showed differences between these enzymes. The analysis also revealed that the MGAT2 and AWAT genes may have arisen from DGAT2 duplication events.

**Conclusions:**

In this study, we identified several DGAT1 and DGAT2 homologs in eukaryote taxa. Overall, the data show that DGAT1 and DGAT2 are present in most eukaryotic organisms and belong to two different gene families. The phylogenetic and evolutionary analyses revealed that DGAT1 and DGAT2 evolved separately, with functional convergence, despite their wide molecular and structural divergence.

## Background

Triacylglycerols (TAGs), fatty acyl ester derivatives of glycerol, are a class of neutral lipids that represent the most important storage form of energy for eukaryotic cells [1,2]. In a number of plant species, TAGs are major storage lipids that accumulate in developing seeds, petals, pollen grains, and fruits [3]. Plant oils have been used for human consumption and have become important renewable resources as biofuels [4,5]. The enzymatic machinery for the formation of TAGs is located in the endoplasmic reticulum (ER). TAGs can then accumulate as oil droplets in the cytoplasm or in specialized oil storage bodies [6], which are generated through budding of the outer ER membrane [7]. A substantial part of TAG synthesis is performed by enzymes of the Kennedy pathway, which sequentially transfer acyl chains from acyl-CoAs to sn-1, -2 and -3 positions of glycerol backbones [8,9]. DGAT (acyl-CoA: diacylglycerol acyltransferase; EC 2.3.1.20) is a transmembrane enzyme that functions in the final step of TAG biosynthesis, catalyzing the acylation of sn-1,2-diacylglycerol (DAG) at the sn-3 position using an acyl-CoA substrate. DGAT has been proposed to be the rate-limiting enzyme in plant storage lipid accumulation [10,11]. Consequently, DGAT is considered a key enzyme for biotechnological purposes; it might be utilized to increase oil content in oleaginous plant species [12-15]. DGAT overexpression causes a net increase in seed oil content in *Arabidopsis *[16]. Additional evidence is also available from studies of *Glycine max *[17], *Brassica napus *[18] and *Zea mays *[19]. TAG can also be formed by an acyl-CoA-independent pathway. In this pathway, the phospholipid:diacylglycerol acyltransferase (PDAT; EC 2.3.1.158) enzyme responsible for TAG synthesis, using phosphatidylcholine (PC) as the acyl donor, in which the transfer of an acyl group from the sn-2 position of PC to the sn-3 position of DAG yields TAG and sn-1 lyso-PC [20-22].

Different types of DGAT enzymes have been identified in several species. DGAT1 and DGAT2 are two of the enzymes that are responsible for the main part of TAG synthesis in most organisms, and they have been studied in many eukaryotic organisms. Another group of DGAT enzymes is represented by the bifunctional DGAT/wax ester synthase (ADP1) from *Acinetobacter calcoaceticus *[23]. Homologs of ADP1 have also been characterized in *Petunia *[24] and *Arabidopsis *[25]. A soluble form of DGAT has been identified in peanut cotyledons [26], and in *Euonymus alatus*, a distinct type of DGAT was recently identified that is responsible for the synthesis of 3-acetyl-1,2-diacyl-sn-glycerols (acTAG), an unusual triacylglycerol [27]. The first cloned DGAT gene was isolated from mice; it corresponds to a member of the DGAT1 family, which has high sequence similarity with the sterol:acyl-CoA acyltransferase (ACAT; EC: 2.3.1.26). DGAT1 is structurally related to the ACATs, with the divergence in its amino acid sequence conferring its substrate specificity to DAG. Both enzymes belong to a large family of membrane-bound O-acyltransferases (MBOAT) [28,29]. The DGAT2 gene was initially identified in the oleaginous fungus *Morteriella ramanniana *[30]. In addition to DGAT2, this family includes acyl-CoA:monoacylglycerol acyltransferase-1 EC:2.3.1.22 (MGAT1) [31], MGAT2 [32,33], MGAT3 [34], and acyl-CoA wax-alcohol acyltransferase (AWAT, EC:2.3.1.75) [35,36]. Interestingly, DGAT2 enzyme family members do not show DNA or protein sequence similarities with DGAT1. Furthermore, DGAT1 proteins are larger than DGAT2 and possess 6 to 9 transmembrane domains, while DGAT2 has only one or two [2]. Thus, it has been suggested that DGAT1 and DGAT2 play non-redundant roles in different tissues and species in TAG synthesis [37-40]. Homologs of the DGAT1 and DGAT2 genes have been identified in several eukaryotic organisms [15,30,36,41-49]. A study of comparative genomics and proteomics in vertebrates suggested that the DGAT1 and DGAT2 gene families have evolved separately during vertebrate evolution [50]. Despite many studies of the DGAT1 and DGAT2 gene families, which emphasize the importance of these enzymes in biotechnology for increasing the accumulation of triacylglycerols [16,51,52], little is known about their evolutionary origins in eukaryotes apart from vertebrates.

The increasing availability of whole genome sequences and the annotation of genes from a wide range of phyla enable deeper phylogenetic analysis of gene families to provide important contextual insight into their present day diversity and evolution. The goal of this study was to examine the evolutionary relationship of the DGAT1 and DGAT2 genes across eukaryotic organisms in order to determine their origin and to verify if these genes evolved separately in eukaryotic organisms. We performed a comparative analysis integrating phylogenetic, computational and structural approaches to the DGAT1 and DGAT2 genes, including analysis of their relationship with the ACAT, MGAT and AWAT genes in a large number of fully sequenced eukaryotic genomes, representing a broad array of taxonomic groups. Most genomes contain both the DGAT1 and DGAT2 genes, though DGAT1 is absent in yeast and basidiomycetes. Our findings suggest that these two enzymes evolved separately since the emergence of eukaryotes and may have different origins.

## Methods

### Data sources and sequence retrieving

DGAT1 and DGAT2 gene and protein sequences were obtained by performing blast searches (blastp, blastx and tblastx) of the Protein and Genome databases with the default parameters and an e-value threshold of 1.0 E-50 at the NCBI (National Center for Biotechnology Information-http://www.ncbi.nlm.nih.gov) and the completed genome projects database at the JGI (Joint Genome Institute-http://www.jgi.doe.gov). The cDNA sequences encoding DGAT1 and DGAT2 from *Arabidopsis *(AT2G19450 and AT3G51520) were used as queries in the blastx and tblastx programs to search for DGAT1 and DGAT2 from different plant species. The predicted peptides were further used in blastp analysis to double check their predicted identities. The mouse DGAT1-2 (NM_010046 and NM_026384), ACAT1-2 (NP_033256 and NP_666176), MGAT1-2 (NM_026713 and NM_177448) and AWAT1-2 (NM_001081136 and NM_177746) cDNA sequences were used as queries in the blastx and blastp to search for homologous sequences. The genome databases of yeast http://www.yeastgenome.org/, fungi and insects http://genome.jgi-psf.org/ were also analyzed using a methodology similar to that described for the plant and vertebrate sequences. Sequences from representative eukaryotic species belonging to plant monocotyledons (*Oryza sativa, Sorgum bicolor, Brachypodium distachyon*), eudicots (*Arabidopsis thaliana, A. lyrata, Brassica napus, Ricinus comunis, Manihot esculenta, Populus trichocarpa, Prunus persica, Medicago truncatula, Glycine max, Vitis vinifera, Cucumis sativus, Mimulus guttatus*), mosses (*Selaginella moellendorfii, Physcomitrella patens*), microalgae (symbiotic *Chlorella *sp., free-living *Coccomixa *sp.), mammals (*Homo sapiens, Pan troglodytes, Bos taurus, Mus musculus, Rattus norvegicus*), bird (*Gallus gallus*), amphibian (*Xenopus tropicalis*), fish (*Danio rerio*), invertebrates (*Apis mellifera, Drosophila melanogaster*), yeast (*Saccharomyces cereviseae, Candida albicans*), fungi (*Aspergillus niger, Alternaria brassicicola, Laccaria bicolor, Schizophylum commune, Agaricus bisporus*) and Amoebozoa (*Dictyostelium discoideum*) were selected. Additional File 1 provides a detailed description of the proteins used and the corresponding accession numbers. *Taxa *terminologies are abbreviated using the first letter of the genus and two letters of the species name (e.g., Ath corresponds to *Arabidopsis thaliana*).

### Sequence Alignments

Nucleotide and protein sequences were aligned using the Alignment Explorer/CLUSTALW [53] implemented in Molecular Evolutionary Genetics Analysis (MEGA version 4.0) [54]. The multiple alignments were manually inspected and edited, and only positions unambiguously aligned were included in the final analysis. The final dataset included a total of 151 sequences from 43 species and 344 amino acid positions. Because the DGAT2 gene was first identified in the oleaginous fungus *Morteriella ramanniana *[30], we also included these sequences in the analysis. In *Tropaeolun majus*, DGAT1 seems to be the sole acyl-CoA-dependent DGAT [15], and this sequence was included in the analysis. We also included in this dataset sequences of other types of DGAT enzymes to compare with DGAT1 and DGAT2, such as the WSD protein sequence from *Arabidopsis *[25] and *Acinetobacter *sp. [55], soluble DGAT from *Arachis hypogaea *[26] and a distinct DGAT (DAcT) from *Euonimus alatus *[27].

### Phylogenetic and structural analyses

The phylogenetic analysis was reconstructed after protein sequence alignments using three different and independent approaches: the neighbor-joining (NJ), the Bayesian and the Maximum-likelihood (ML) methods. The NJ method was performed with MEGA 4.0 [54]. The molecular distances of the aligned sequences were calculated according to the poisson correction model. All gap and missing data in the alignments were accounted for by pairwise deletion. Branch points were tested for significance by bootstrapping with 1000 replications. The ML analysis was performed using PhyML3.0 [56]. Branch points were tested for significance by bootstrapping with 100 replicates. Bayesian analysis was conducted in MrBayes 3.1.2 [57] with the mixed amino acid substitution model plus gamma and invariant sites. Two independent runs of 10,000,000 generations each with two Metropolis-coupled Monte Carlo Markov chains (MCMCMC) were run in parallel (starting each from a random tree). Markov chains were sampled every 100 generations, and the first 25% of the trees were discarded as burn-in. The remaining ones were used to compute the majority rule consensus tree (MrBayes command allcompat), the posterior probability of clades and branch lengths. Convergence of the two runs was assessed by checking the average standard deviation of split frequencies (below 0.01) and the Potential Scale Reduction Factor (PSRF, very close to 1.00 for all parameters).

The structural organization of the DGAT genes was determined after alignment of genomic DNA and cDNA and EST sequences. Genomic sequences were also analyzed in the FGENESH gene structure prediction program http://www.softberry.com/[58]. Predicted transmembrane structures were obtained using the transmembrane prediction server TMHMM-2.0 http://www.cbs.dtu.dk/services/ and SMART database http://smart.embl-heidelberg.de/ with the complete protein sequences.

### Divergence estimates

Pairwise synonymous (*Ks*) and non-synonymous (*Ka*) numbers of substitutions corrected for multiple hits were calculated using the DnaSP software (DNA polymorphism analysis) [59].

### Cloning and Yeast Complementation

Leaves from Castor bean (*Ricinus communis cv*.EBDA-MPB1) two month-old plants were used for total RNA extraction using the Nucleospin RNA Plant kit (Macherey-Nagel) following the manufacturer's instructions. First strand cDNA synthesis was performed with oligo(dT) primers and M-MLV reverse transcriptase (Promega). The complete open reading frames from castor RcDGAT1 (AY366496) and RcDGAT2 (EU391592) genes were amplified using Pfx50 polymerase (Invitrogen) with the following primers: 5'-CACCTCTAGAATGACGATTCTCGAAACGCC-3' and 5'-CTCGAGTCAGTTCCCATCGCGATTCATT-3 for RcDGAT1 and 5'-CACCTCTAGAATGGGGGAAGAAGCGAATCA-3' and 5'-CTCGAGTCAAAGAATTTCAAGTGTAAGGTCTGC-3' for RcDGAT2. *Xba*I and *Xho*I sites added for subsequent manipulations are underlined. The resulting blunt ended PCR fragments were cloned into pENTR/D-TOPO (Invtrogen) following the manufacturer's instructions. For complementation experiments in yeast, the cloned cDNA fragments were transferred to pVT103-U [53] as *Xba*I/*Xho*I inserts, and the following resulting plasmids were introduced into yeast (*Saccharomyces cerevisiae*) strains [60]: *are1 are2 *mutant (H1112), and a quadruple mutant strain *are1 are2 lro1 dga1 *(H1246, kindly donated by Dr. Sten Stymne, Swedish University of Agricultural Sciences).

### Yeast growth and lipid analysis

Single colonies of vector-only, RcDGAT1 or RcDGAT2 transformed yeast in the H1112 and H1246 mutant backgrounds were inoculated in 10 mL of synthetic complete medium with Ura omitted and grown for 72 h at 28°C. Cultures were centrifuged for 4 min at 10,000 g. Cell pellets were twice extracted with 1.0 mL of chloroform-methanol solution (10:1, v/v) after intense agitation for 2 min with 0.1 mm glass-beads. The chloroform phase was collected and evaporated under N_2 _flow at room temperature. Lipids were analyzed by thin-layer chromatography (TLC) on silica-gel 60 (TLC Aluminium Sheets, Merck, Darmstadt) using the hexane/diethyl ether/acetic acid (90:10:1 v/v) solvent system, which is specific for resolving neutral lipids [61]. Lipids were revealed after staining with a 0.03% solution of Coomassie Brilliant Blue R250 (Sigma) in 20% methanol [62]. Triolein, cholesterol and cholesterol-ester reference lipids, used for TLC, were all obtained from Sigma.

## Results

### Identification of DGAT homologous sequences

We have conducted a broad survey of fully sequenced genomes, including representatives of Amoebozoa, yeasts, fungi algae, mosses, plants (monocots and eudicots), vertebrate (mammalian, fish and amphibian) and invertebrate (insect), for the presence of DGAT1 and DGAT2 gene homologs. The full list of genes resulting from this analysis is summarized in Additional File 1. A set of 36 completely sequenced and 7 non-sequenced genomes were investigated. A total of 151 sequences were identified, of which 91 were DGATs (DGAT1 and DGAT2), 56 corresponded to ACAT, MGAT and AWAT and the remaining were sequences of other types of DGATs. These other DGAT types include a soluble DGAT from peanut, a distinct DGAT (DAcT) from *Euonymus alatus *and a bifunctional DGAT/wax synthase (WSD) from *A. thaliana *and *Acinetobacter *sp.

It was verified that the DGAT1 and DGAT2 genes are almost ubiquitously found in eukaryotes, being readily identifiable in all the major eukaryotic groups and in all genomes examined with the notable exception of yeast (*S. cereviseae *and *Candida albicans*) and Basidiomycetes fungi (*Laccaria bicolor, Schizophillum commune *and *Agaricus bisporus*), which do not contain a DGAT1 gene. Most organisms possess a single copy of each DGAT gene. The remarkable exception to this general pattern occurs in some members of green plants, revealing the possible existence of lineage-specific duplications. Homologs to ACAT genes were found in yeast, fungi, vertebrates (mammalian, fish and amphibian) and invertebrates (insects) but not in plants. The MGAT and AWAT genes were found only in vertebrate taxa. In Amoebozoa species (*Dictyostelium discoideum*), four putative DGAT sequences were identified.

### Evolutionary relationship of the DGAT gene family in Eukaryotes

**Figure 1:**
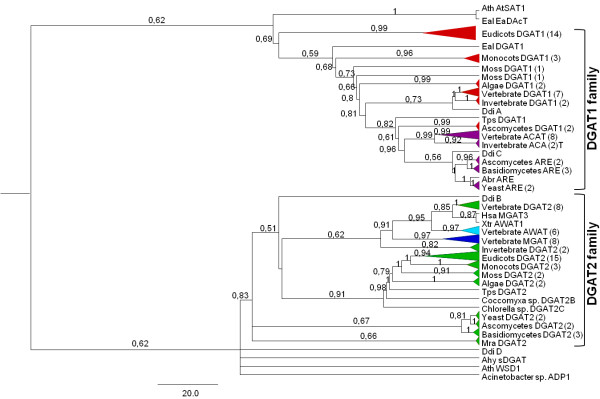
**Phylogenetic relationships between DGAT1 and DGAT2 gene families reconstructed by the Bayesian method**. A total of 151 protein sequences from eukaryotic species and 344 sites were included in the analyses. The posteriori probabilities are labeled above the branches. Only values higher than 0.5 are presented. Numbers within brackets correspond to the number of species within each group. The enzymes are represented by different colors in the phylogenetic tree: DGAT1 (red), DGAT2 (green), ACATs (purple), MGAT (dark blue) and AWAT (light blue). *Taxa *terminologies are abbreviated using the first letter of the genus and two letters of the species name: *Alternaria brassicicola *(Abr), *Arabidopsis thaliana *(Ath), *Dictyostelium discoideum *(Ddi), *Euonymus alatus *(Eal), *Homo sapiens *(Hsa), Mortierella ramanniana (Mra), *Thalassiosira pseudonana *(Tps), *Xenopus tropicalis *(Xtr).

The 151 eukaryote DGAT amino acid sequences and related ACAT, MGAT and AWAT enzymes were used for the reconstruction of phylogenetic trees. A total of 344 positions were included in the final dataset. Phylogenetic analyses of the DGAT1 and DGAT2 amino acid sequences resulted in a well-resolved tree. Results from Bayesian NJ and ML tree analysis produced similar topologies and revealed evolutionary partitioning of the DGAT protein family into two major clades: DGAT1 and DGAT2 (Figure 1 and Additional Files 2 and 3). Vertebrate and invertebrate ACAT1-2 and yeast and fungi ARE proteins grouped within the DGAT1 clade (Figure 1 and Additional Files 2 and 3). The MGAT and AWAT protein grouped into the DGAT2 clade. Although the ACAT and DGAT1 proteins are closely related in phylogenetic trees, the ACAT sequences from yeast, fungi, vertebrates and invertebrates clustered separately from DGAT1 sequences. Interestingly, DGAT2 sequences from vertebrates do not form a monophyletic group with the DGAT2 homolog sequences from yeast, fungi, invertebrates and plants. Instead, the DGAT2 sequences from vertebrates appear more closely related to the vertebrate MGAT2 and AWAT clades, suggesting that these genes may have arisen from DGAT2 duplication events. Inside both DGAT1 and DGAT2 clades from plants, monocotyledons and eudicots grouped separately. Two of the four DGATs sequence homologs found in Amoebozoa (denominated A and B) grouped into the DGAT1 and DGAT2 clade, respectively, suggesting that DGAT1 and DGAT2 diverged very early in eukaryotic evolution. EaDAcT (*E. alatus*), AtSAT (*A. thaliana*), a soluble DGAT from peanut and WSD from *A. thaliana *and *Acinetobacter *sp. grouped separately from DGAT1 and DGAT2, but with low bootstrap and posteriori probability values. This result indicates that these types of DGAT enzymes are phylogenetically divergent from DGAT1 and DGAT2. Genes encoding EaDAcT, SAT, WSD and soluble DGAT enzymes have not yet been identified in other organisms.

### Structural organization and transmembrane domain prediction

**Figure 2:**
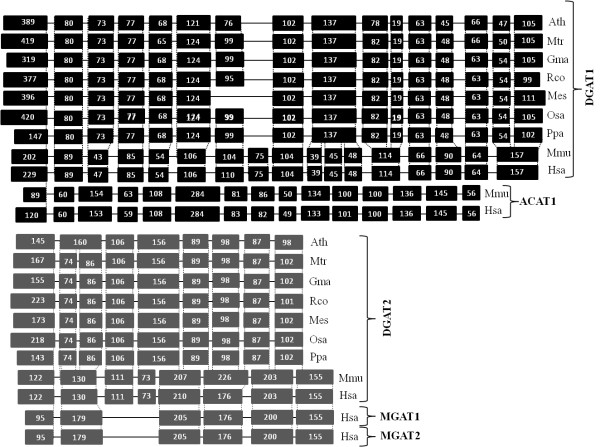
**Structural organization of plant DGAT1 and DGAT2 genes and vertebrate ACAT, MGAT and AWAT**. Exon sequences are represented as simplified boxes. The size of each exon (in bp) is given. Bars represent introns. Black boxes represent DGAT1 and ACAT exons and grey boxes represent DGAT2 and MGAT exons. *A. thaliana *(Ath), *M. trucatula *(Mtr), *G*. *max *(Gma), *R. comunis *(Rco), *M. esculenta *(Mes), *O. sativa *(Osa), *P. pattens *(Ppa), *M. musculos *(Mmu) and *H. sapiens *(Hsa).

To further investigate the relationships between the DGAT1 and DGAT2 gene families, we compared the structural organization of the DGAT genes, when gene and corresponding cDNA were available. Comparisons were performed between DGAT1 and DGAT2 genes from plants and vertebrates (mouse and human). A detailed comparison of DGAT1 and DGAT2 genes across plant species and vertebrates (Figure 2) revealed a high degree of conservation in gene structure within each type of DGAT (DGAT1 and DGAT2). However, the structure of the genes encoding the DGAT1 protein is distinct from that of the DGAT2 gene. The DGAT1 genes harbor 15 to 17 exons, while DGAT2 genes have 8 to 9 exons. In higher plants, DGAT1 proteins are encoded by genes that are generally comprised of 16 exons and 15 introns (Figure 2). An exception to this pattern was observed for *M. esculenta*, which probably lost its 7th exon and has 15 exons. The DGAT2 genes are generally comprised of 9 exons, with the exception of *Arabidopsis*, which has 8. The vertebrate ACAT has 15 exons, while MGAT has 8 exons. Another important finding was that the structures of the DGAT1 and ACAT genes are more related to each other than to the structure of genes encoding DGAT2, which is more related to the MGAT gene (Figure 2).

**Figure 3:**
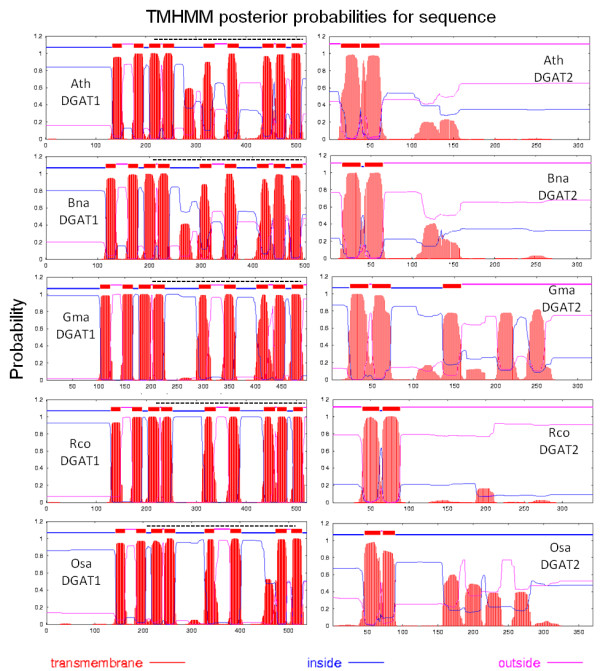
**Predicted transmembrane domain for plants *A. thaliana *(Ath), *B. napus *(Bna), *G. max *(Gma), *M. truncatula *(Mtr), *R. comunis *(Rco), *P. trichocarpa *(Ptr), *O. sativa *(Osa), and *Z. mays *(Zma) DGAT1 and DGAT2 sequences**. See Additional File 1 for sources. The TMHMM web tools of the Center for Biological Sequence Analysis, Technical University of Denmark TMHMM Server plots the probability of the ALDH sequence forming a transmembrane helix (0-1.0 on the y-axis) (shown in red for the relevant amino acid sequences) http://www.cbs.dtu.dk/services/TMHMM/. Eight and nine predicted transmembrane helices are identified for DGAT1 sequences, while two to four transmembrane helix (or helices) were observed for DGAT2 sequences; regions of all sequences predicted to be located inside or outside the membrane are shown in blue and pink, respectively. The Pfam:Mboat is represented by a dotted line to DGAT1, ACAT1 and ACAT2.

**Figure 4:**
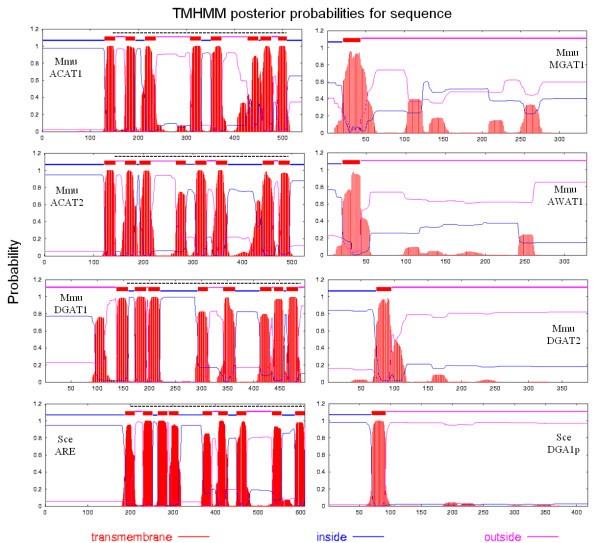
**Predicted transmembrane domain for Mouse (Mmu) DGAT1, DGAT2, ACAT1, ACAT2, MGAT1, and AWAT1 sequences**. See Additional File 1 for sources. The TMHMM web tools of the Center for Biological Sequence Analysis, Technical University of Denmark TMHMM Server plots the probability of the ALDH sequence forming a transmembrane helix (0-1.0 on the y-axis) (shown in red for the relevant amino acid sequences) http://www.cbs.dtu.dk/services/TMHMM/. Eight predicted transmembrane helices are identified for each of the DGAT1, ACAT1 and ACAT2 sequences; in contrast, only one major transmembrane helix (or helices) was observed for DGAT2, MGAT1, MGAT2, AWAT1 and AWAT2 sequences; regions of all sequences predicted to be located inside or outside the membrane are shown in blue and pink, respectively. The Pfam:Mboat is represented by a dotted line to DGAT1, ACAT1 and ACAT2.

Predictions of transmembrane (TrM) structures were performed, and comparisons were made between plant DGAT1 and DGAT2 and mouse DGAT1, DGAT2, ACAT, MGAT and AWAT (Figure 3 and 4). For each plant DGAT1 subunit examined, 8 and 9 regions containing highly probable TrM sequences were predicted for monocotyledons and eudicots, respectively. For DGAT2, only 2 regions containing highly probable TrM sequences were detected in both monocotyledons and eudicots (Figure 3). The comparisons among mouse DGAT1, DGAT2, ACAT, MGAT and AWAT subunits revealed that DGAT1 is more closely related to ACAT, which has 8 high probable TrM regions, while DGAT2 is more closely related to the MGAT and AWAT proteins, with only 1 TrM region. Interestingly, *S. cerevisae *acyl-CoA sterol acyltransferase (coded by the ARE gene) includes 9 TrM regions, as found in plant DGAT1 and in mouse DGAT1 and ACAT. Further, *S. cereviceae *Dga1 (homologue to DGAT2) possesses only one TrM region, as found in plant DGAT2 and mouse DGAT2, MGAT and AWAT (Figure 4). Moreover, both ACAT and DGAT1 proteins have an MBOAT (membrane bound O-acyltransferase) domain (Figure 3 and 4), while DGAT2, MGAT and AWAT do not. These results support the hypothesis that DGAT1 and DGAT2 belong to different gene families and evolved separately during eukaryote evolution, as is demonstrated by the phylogenetic tree. Additional Files 4 and 5 show the multiple sequence alignment of DGAT1 and DGAT2 protein sequences, respectively. In these alignments, we observed similarities between DAGT1 and ACAT and DGAT2 with MGAT and AWAT protein sequences.

### Divergence estimates

A comparison of the rate of non-synonymous substitutions (*K_a_*) to the rate of synonymous substitutions (*K_s_*) was performed; such comparison can be used as an indicator of selective pressure acting on a protein-coding gene [63,64]. We calculated *K_a_*/*K_s_*ratios for vertebrate DGAT1, DGAT2, ACAT, MGAT and AWAT and for plant DGAT1 and DGAT2. From these analyses it can be noted that distinct selective pressures have been operating on the DGAT1 and DGAT2 genes, as different rates were obtained (Additional file 6 and 7). Comparisons between vertebrate DGAT1 and ACAT showed high *K_a_*/*K_s_*ratios, while low *K_a_*/*K_s_*ratios were observed between DGAT2 with MGAT and AWAT from vertebrates (Additional file 6). The comparisons between DGAT1 and DGAT2 in both vertebrates and plants revealed a high level of *K_a_*/*K_s_*ratios (Additional file 6 and 7). We also compared DGAT1 and DGAT2 between eudicots and monocots and between oilseed and no oilseed plants and verified low *K_a_*/*K_s_*ratios, indicating that they are under purifying selection.

### DGAT1 and DGAT2 and triacylglycerol biosynthesis

**Figure 5:**
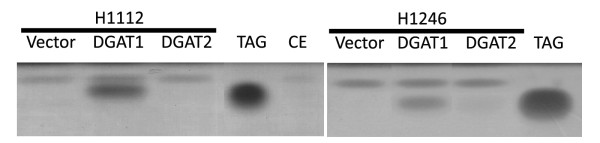
**Evaluation of triacylglyceride biosynthesis in yeast mutants complemented with castor bean DGAT1 or DGT2**. The H1122 strain is an *are1 are2 *double mutant, while H1246 is an *are1 are2 Iro1 dga1 *quadruple mutant. Lipids were separated in a silica-gel plate of thin-layer chromatography, and the separation range corresponding to Triacylglicerol (TAG) and ADAG (mono-alkyl-diacylglicerol), used as lipid standards, is shown after Coomassie-Blue R250 staining.

Two different yeast mutants that are defective in TAG biosynthesis were used to determine if both castor bean DGAT orthologs are able to complement the mutated enzymes and allow the synthesis and accumulation of TAG (Figure 5). TAG synthesis was undetectable in both mutant strains H1112 (*are1 are2*) and H1246 (*are1 are2 lro1 dga1*) carrying the empty expression vector. Only DGAT1 was able to reestablish TAG synthesis in the H1112 strain and H1246 yeast mutants. The equivalence of lipid amounts among samples was confirmed by the homogeneous amount of mono-alkyl-diacyl-glycerol.

## Discussion

It has long been understood that DGAT enzymes play important roles in triacylglycerol biosynthesis. DGAT is the only enzyme in the pathway that is thought to be exclusively committed to TAG synthesis, and thus it is considered a key enzyme in this reaction [10,13,16]. DGAT activity was first reported in 1956 [65], but it was only in the last decade that DGAT genes were cloned and molecular tools for studying TG synthesis became available [2]. Although many studies have been conducted with these enzymes, little is known about their evolutionary origins. Taking advantage of the wide availability of data on the genomes of several species, this study focused on the characterization of the origin and evolutionary history of the DGAT1 and DGAT2 gene families in eukaryotes. Our survey of DGAT1 and DGAT2 proteins shows that they are present in all eukaryotes searched, with the exception of yeast (*Saccharomyces cereviseae *and *Candida albicans*) and basidiomycetes (*Laccaria bicolor, Schizophyllum commune *and *Agaricus bisporus*), which lack DGAT1 (Figure 1 and Additional Files 1, 2 and 3). This demonstrates that the DGAT1 and DGAT2 genes encode essential enzymes for most eukaryotic organisms. In the yeast *S. cereviseae*, two genes that have homology with DGAT1 were identified, ARE1 and ARE2, which encode ACAT-related enzymes. The yeast enzymes are 49 percent identical to each other and exhibit 23 percent identity to human ACAT [66]. Both these genes have been suggested to be involved in the synthesis of sterol esters. ARE1 is also involved in the synthesis of TAG, whereas the ARE2 gene is more specifically involved in the synthesis of sterol esters [67]. Insights into the functions of DGAT1 and DGAT2 in triglyceride metabolism have been provided by studies in yeast. With gene knockout or overexpression studies, several groups have demonstrated that the yeast DGA1 (DGAT2 homologue), is the major DGAT enzyme contributing to triglyceride synthesis and storage in yeast [60,68,69]. However, it has been demonstrated with genetic complementation that DGAT1 from *Arabidopsis thaliana *can restore TAG synthesis to the yeast dga1:lro1:are1:are2 quadruple mutant, which is devoid of TAG and sterol esters [70]. In the present work, using the castor bean genes, we obtained similar results with DGAT1, and were also unable to demonstrate DGAT2 ability to complement TAG synthesis compared to DGAT1, as previously described by Zhang and collaborator's using DGAT2 from Arabidopsis [70]. This indicates that despite their apparent convergent functional evolution, DGAT1 and DGAT2 have different affinities for identical substrates, and their effective activity with accumulation of TAG depends on how much the other enzymes from the same pathway consume or provide DGAT substrate and precursors. The absence of DGAT1 genes in yeast and in basidiomycetes indicates that TGAs are not essential in these organisms and that other enzymes may be able to produce the required amount of triacylglycerol. Mutant *S. cerevisiae *strains devoid of DGAT activity and lacking triglycerides are viable and grow normally, indicating that triglycerides are not essential for the survival of this yeast [60]. However, a recent study showed that triglyceride synthesis is essential for viability of the fission yeast *Schizosaccharomyces pombe *[71].

Our phylogenetic analysis confirmed the existence of separate clades for DGAT1 and DGAT2 genes in eukaryotes, indicating that they evolved asymmetrically. In the phylogenetic analysis, DGAT1 was more closely related and grouped with ACAT genes. ACAT is involved in cholesterol ester biosynthesis in mammals [28], and homologs of these genes were found in fungi, insects and vertebrates but not in plants. We verified a conserved motif between DGAT1 and ACAT (FYXDWWN; amino acids 392-398 of *A. thaliana *DGAT1) in all organisms analyzed that has also been reported in other studies [43,72]. This motif has been implicated in the binding of fatty acyl-CoA, a common substrate for these enzymes [72]. Additionally, DGAT2 shares homology and groups with acyl-CoA: monoacylglycerol acyltransferases [31,73]. Topological studies indicated that the majority of DGAT2 is composed of a C-terminal cytoplasmic domain. The amino acids HPHG make up residues 161-164 of murine DGAT2, suggesting that this sequence plays a crucial role in the function of the enzyme and may be part of the active site [74]. The HPHG-motif is conserved in DGAT2 in yeast, fungi, invertebrates and vertebrates and is also present in MGAT and AWAT. Plant DGAT2 contains a corresponding sequence of conserved amino acids, but the motif is modified to EPHSV (108-112 of *A. thaliana *DGAT2), which is conserved in all plant species analyzed. Three DGAT2 homologs were found in the microalgae *Chlorella *sp. (chrDGAT2A, chrDGAT2B and chrDGAT2C). In NJ and ML analysis, chrDGAT2C is more related with vertebrate, invertebrate, fungi and yeast DGAT2 members and also with the MGAT and AWAT proteins. chrDGAT2C has an HPHG motif and chrDGAT2A and chrDGAT2B have EPHSV motifs, similar to the plant DGAT2. The same pattern was found in two unicellular photoautotrophic green algae, *Ostreococcus tauri *and *O. lucimarinus*, which have three DGAT2 genes. One of them shows significant homology to mammalian MGAT, whereas the other two seem to be more closely related to plant DGAT2 members [75]. This suggests that DGAT2 in these organisms may have evolved by different duplication events.

**Figure 6:**
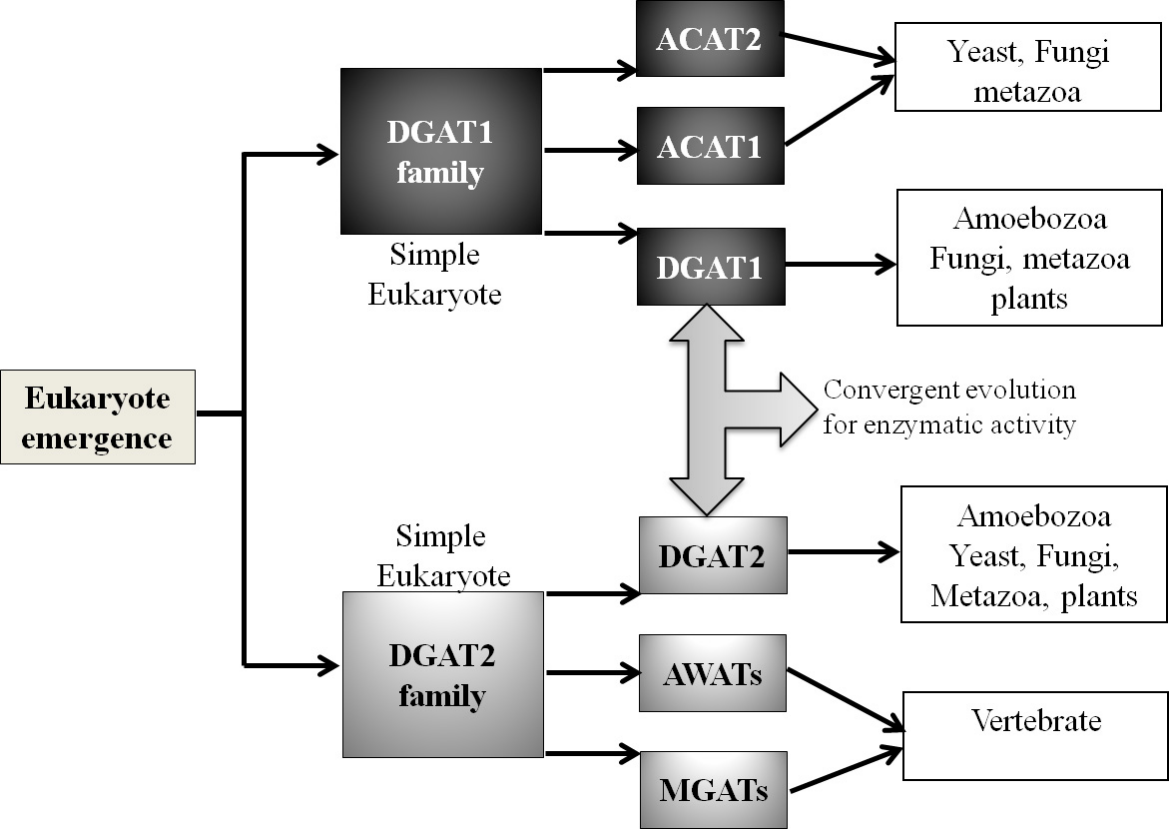
**Scenarios proposed for the evolution of DGAT1 and DGAT2 in eukaryotes**. The proposed evolutionary relationships between DGAT1 and DGAT2 family members are as follows: DGAT1 and DGAT2 have evolved asymmetrically since the eukaryotic emergence and underwent convergent evolution likely due to the importance of TAGs for all eukaryotic organisms.

Notably, the DGAT1 and DGAT2 families arose from different ancestors during the emergence of eukaryotes, and our results suggest that they followed convergent evolution in eukaryotes despite having evolved separately since the early eukaryotes, as depicted in the model in Figure 6. The high sequence similarity and close phylogenetic relationship of the DGAT1 and ACAT genes, which is higher than that of DGAT1 and DGAT2, strongly suggests that DGAT1 and ACAT emerged from a common ancestral sequence, whereas DGAT2 emerged from another ancestral sequence. We propose that the DGAT1 and DGAT2 genes had a functional convergence in eukaryotes, which may be explained by the great importance of DGAT activity to the formation of TAGs in all eukaryotes. In several species, the formation of TAGs plays important roles not only as a storage reserve but also in growth and development. In agreement with our findings, it has been proposed that the DGAT1 and DGAT2 enzymes have different biochemical properties and physiological functions. In overexpression studies in mice, DGAT2 was identified as a more active enzyme than DGAT1, yielding a higher increase in intracellular triacylglycerols that accumulated as large, centrally located, cytosolic droplets [76]. Mice lacking DGAT1 (Dgat1^-/- ^mice) are viable and have modest reductions in tissue triacylglycerols [77], whereas mice lacking DGAT2 (Dgat2^-/- ^mice) have severe reductions in whole body triacylglycerols and die shortly after birth [76]. In *A. thaliana*, mutation of the DGAT1 gene severely affects seed development, causing the appearance of wrinkled and incompletely filled seeds, aberrant seedling growth, reduced seed oil content, an increased DAG/TAG ratio and the beta-oxidation of elevated fatty acid (FA) levels [13,15]. It has been also demonstrated that DGAT1, but not DGAT2, is a multifunctional acyltransferase that catalyzes the synthesis of diacylglycerol, retinyl esters, and waxes in addition to triacylglycerol in *in vitro *assays [78]. DGAT1 cannot compensate for the absence of DGAT2, highlighting the functional differences between these enzymes. Furthermore, compared to DGAT1, DGAT2 appears to be more active at lower concentrations of acyl-CoA (≤ 50 μM) and less active at concentrations below 50 mM magnesium [38]. The two enzymes may also differ in terms of substrate specificities. Substrate specificity assays using a mammalian DGAT2 expressed in insect cells showed that oleoyl-CoA was utilized preferentially, followed by palmitoyl-CoA, while activity levels with linoleoyl- and arachidonyl-CoA were similar [38]. However, in enzymatic assays using soluble and non-soluble samples of the fungal DGAT2 from *M. ramanniana*, no significant preference was detected between 12:0-CoA and 18:1-CoA as an acyl donor substrate [30]. In plant species, although both DGAT1 and DGAT2 significantly contribute to TAG formation, the relative contribution of each enzyme seems to be species dependent and may also differ in different tissues within the same plant species [40]. Moreover, it was reported that DGAT1 and DGAT2 are located in different areas of the endoplasmic reticulum (ER) [40]. The fatty acid profile of TAG may be determined by distinct substrate specificities of the DGAT enzymes in different species and in plants containing unusual fatty acids. DGAT2 may have a major role in channeling unusual fatty acids into seed storage oils [15,40]. In a recent study with *Ricinus comunis*, it was demonstrated that DGAT2 presents higher mRNA accumulation than DGAT1 during castor seed development [79]. It was also demonstrated in *R. comunis *that only RcDGAT2 can increase hydroxy fatty acid levels in transgenic Arabidopsis, while RcDGAT1 cannot [80]. DGAT2 is more likely to play a major role in seed TAG biosynthesis than DGAT1 in this plant species [44]. In addition, a study of Vernolic acid in *Vernonia galamensis *concluded that both DGAT1 and DGAT2 increase epoxy fatty acid accumulations, but DGAT2 had a greater effect [81]. All these findings corroborate with our results that show that DGAT1 and DGAT2, despite being evolutionarily divergent enzymes, play important roles in TAG metabolism in all eukaryotic organisms. Therefore, we conclude that they are very ancient enzymes that arose quite early in eukaryotic evolution.

The same patterns were observed in structural and transmembrane analysis. The topology models based on prediction algorithms suggested that DGAT1 and ACAT had eight transmembrane domains and 15-17 exons, whereas DGAT2 had 1 or 2 transmembrane domains and 8 exons, similar to MGAT and AWAT. This reinforced the idea that despite their ability to catalyze similar reactions, DGAT1 and DGAT2 belong to different gene families that share neither DNA nor protein sequence similarity [2]. In addition, topological studies show that DGAT1 has its N-terminus oriented towards the cytosol and C-terminal region, which accounts for approximately 50% of the protein, and is present in the ER lumen [82]. Conversely, DGAT2 is an integral membrane protein with both the N and C termini oriented toward the cytosol [74]. In agreement with our results, a comparative genomics and proteomics study of vertebrate DGATs revealed that the predicted secondary and transmembrane (TrM) structures of DGAT1 and DGAT2 were distinct [50]. All of the DGAT2 subunits had one or two predicted TrM(s), whereas DGAT1 was predicted to have nine regions with high probability for TrM [50]. In contrast, a recent study about the membrane topology of murine DGAT1 indicates that DGAT1 has three transmembrane domains [82]. For murine DGAT2, two transmembrane domain were identified [74]. Studies were also carried out in plants and have shown that *Helianthus annuus *[48], *Echium *[83] and *Brassica juncea *[43] have nine transmembrane domains in DGAT1, while DGAT2 from *Brassica juncea *have two transmembrane domains [43].

The Ka/Ks ratios for vertebrate DGAT1, DGAT2, ACAT, MGAT and AWAT and for plant DGAT1 and DGAT2 showed that distinct selective pressures have been operating on the DGAT1 and DGAT2 genes. The comparisons between species groups within of each DAGT1 and DAGT2 family revealed high *K_a_/K_s_*ratios, while low *K_a_/K_s_*ratios were observed between the DGAT1 and DGAT2 families. This indicates a purifying selection within each family with a strong positive selection between them. In the DGAT1 and ACAT genes, in addition to a high level of similarity between them, a positive selection has taken place at multiple locations.

## Conclusions

Overall, our data show that the DGAT1 and DGAT2 proteins are present in most eukaryotic organisms as different gene families. Phylogenetic and evolutionary analyses revealed that DGAT1 and DGAT2 evolved separately with functional convergence since the emergence of eukaryotes and are widely divergent in molecular and structural aspects.

## Authors' contributions

ACTZ retrieved the sequences used in the study, made the sequence alignments and performed the phylogenetics, structural and evolution analyses. FSM contributed to cloning and yeast complementation. RM and CMBA performed the lipid analysis. MMP and RM conceived the study. RM, MMP, GLM, and AC contributed to the analysis interpretation. All authors contributed to the writing of the manuscript and read and approved the final manuscript.

## Supplementary Material

Additional file 1 **The DGAT1, DGAT2, ACAT, MGAT, AWAT and other enzymes used for the analyses**. The table shows the species, gene names and accession numbers of the sequences used in the analyses. Click here for file

Additional file 2 **Phylogenetic tree of DGAT1 and DGAT2 gene families reconstructed by the Neighbor joining (NJ) method**. A total of 151 protein sequences from eukaryotic species and 344 sites were included in the analyses. The bootstrap values are labeled above the branches. Only values > 50% are presented. Numbers within brackets correspond to the number of species within each group. The enzymes are represented by different colors in the phylogenetic tree: DGAT1 (red), DGAT2 (green), ACATs (purple), MGAT (dark blue) and AWAT (light blue). *Taxa *terminologies are abbreviated using the first letter of the genus and two letters of the species name: *Alternaria brassicicola *(Abr), *Arabidopsis thaliana *(Ath), *Dictyostelium discoideum *(Ddi), *Euonymus alatus *(Eal), *Homo sapiens *(Hsa), Mortierella ramanniana (Mra), *Thalassiosira pseudonana *(Tps), *Xenopus tropicalis *(Xtr). Click here for file

Additional file 3 **Phylogenetic tree of the DGAT1 and DGAT2 gene families reconstructed by the Maximum likelihood (ML) method**. A total of 151 protein sequences from eukaryotic species and 344 sites were included in the analyses. The bootstraps values are labeled above the branches. Only values > 50% are presented. Numbers within brackets correspond to the number of species within each group. The enzymes are represented by different colors in the phylogenetic tree: DGAT1 (red), DGAT2 (green), ACATs (purple), MGAT (dark blue) and AWAT (light blue). *Taxa *terminologies are abbreviated using the first letter of the genus and two letters of the species name: *Alternaria brassicicola *(Abr), *Arabidopsis thaliana *(Ath), *Dictyostelium discoideum *(Ddi), *Euonymus alatus *(Eal), *Homo sapiens *(Hsa), Mortierella ramanniana (Mra), *Thalassiosira pseudonana *(Tps), *Xenopus tropicalis *(Xtr). Click here for file

Additional file 4 **Multiple sequence alignment of predicted amino acid sequences of DGAT1 proteins**. DGAT1 sequences from *A. thaliana *(Ath), *M. truncatula *(Mtr), *R. comunis *(Rco), *O. sativa *(Osa), *Z. mays *(Zma) and *M. musculus *(Mmu), and ACAT proteins from *M. musculus *and *S. cerevisieae *(Sce) were aligned. Identical residues are shaded black, and similar residues are shaded gray. The DAG/phorbol ester binding signature motif is underlined with a dotted line; the triangle (▼) shows the conserved phenylalanine. The conserved motif between DGAT1 and ACATs is underlined. Click here for file

Additional file 5 **Multiple sequence alignment of deduced amino acid sequences of DGAT2 proteins**. DGAT2 sequences from *A. thaliana *(Ath), *M. truncatula *(Mtr), *R. comunis *(Rco), *O. sativa *(Osa), *Z. mays *(Zma), *M. musculus *(Mmu) and *S. cerevisieae *(Sce) were aligned with MGAT and AWAT proteins from *M. musculus*. Identical residues are shaded in black, and similar residues are shaded in gray. Click here for file

Additional file 6 **Estimation of Ka/Ks rates on vertebrate sequences**. Substitution rates between full-length cDNA sequences of vertebrate DGAT1, DGAT2, ACAT1, ACAT2, MGAT and AWAT. Comparisons were performed between ACATs and DGAT1, MGATs and DGAT2 and between AWAT and DGAT2. Click here for file

Additional file 7 **Estimation of Ka/Ks rates on plant sequences**. Substitution rates between full-length cDNA sequences of plant DGAT1 and DGAT2. Comparisons were performed for DGAT1 and DGAT2 between monocotyledon and eudicotyledon, between oilseed and non-oilseed plants and between DGAT1 and DGAT2 from all plant species. Click here for file
