# Supplementary figures and images for: Evolutionary view of acyl-CoA diacylglycerol acyltransferase (DGAT), a key enzyme in neutral lipid biosynthesis
Source: BMC Evol Biol. 2011 Sep 20;11:263. doi: 10.1186/1471-2148-11-263 (PMC3185287; doi:10.1186/1471-2148-11-263)

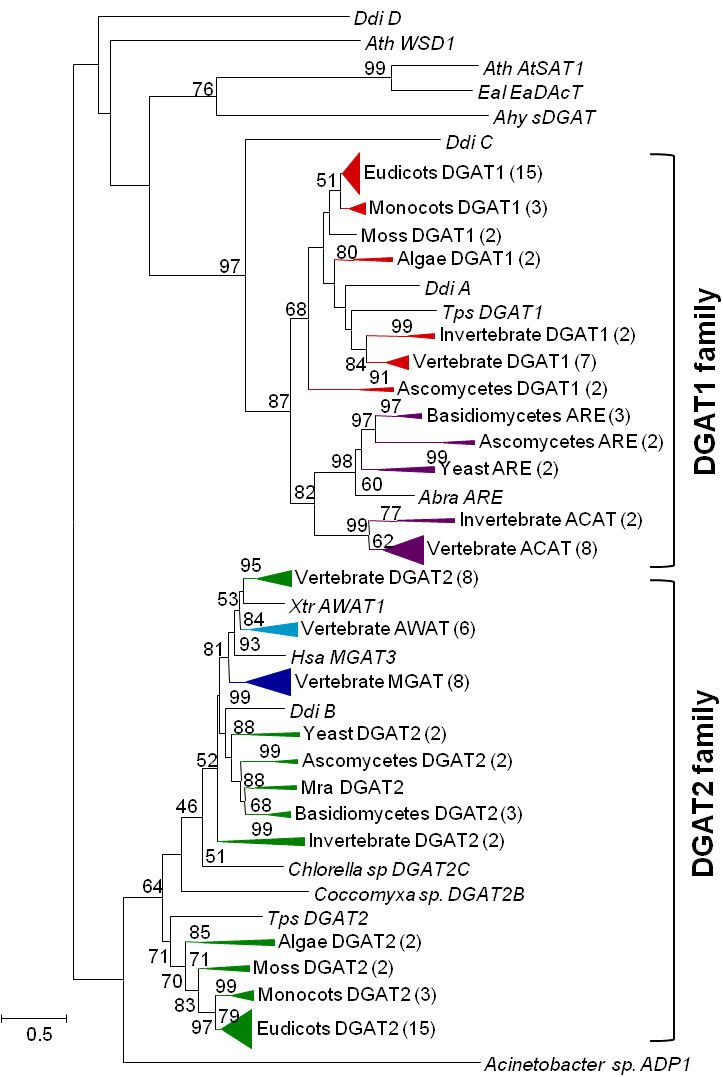

Supplement: Additional file 2 — Phylogenetic tree of DGAT1 and DGAT2 gene families reconstructed by the Neighbor joining (NJ) method. A total of 151 protein sequences from eukaryotic species and 344 sites were included in the analyses. The bootstrap values are labeled above the branches. Only values > 50% are presented. Numbers within brackets correspond to the number of species within each group. The enzymes are represented by different colors in the phylogenetic tree: DGAT1 (red), DGAT2 (green), ACATs (purple), MGAT (dark blue) and AWAT (light blue). Taxa terminologies are abbreviated using the first letter of the genus and two letters of the species name: Alternaria brassicicola (Abr), Arabidopsis thaliana (Ath), Dictyostelium discoideum (Ddi), Euonymus alatus (Eal), Homo sapiens (Hsa), Mortierella ramanniana (Mra), Thalassiosira pseudonana (Tps), Xenopus tropicalis (Xtr). [file 1471-2148-11-263-S2.TIFF]

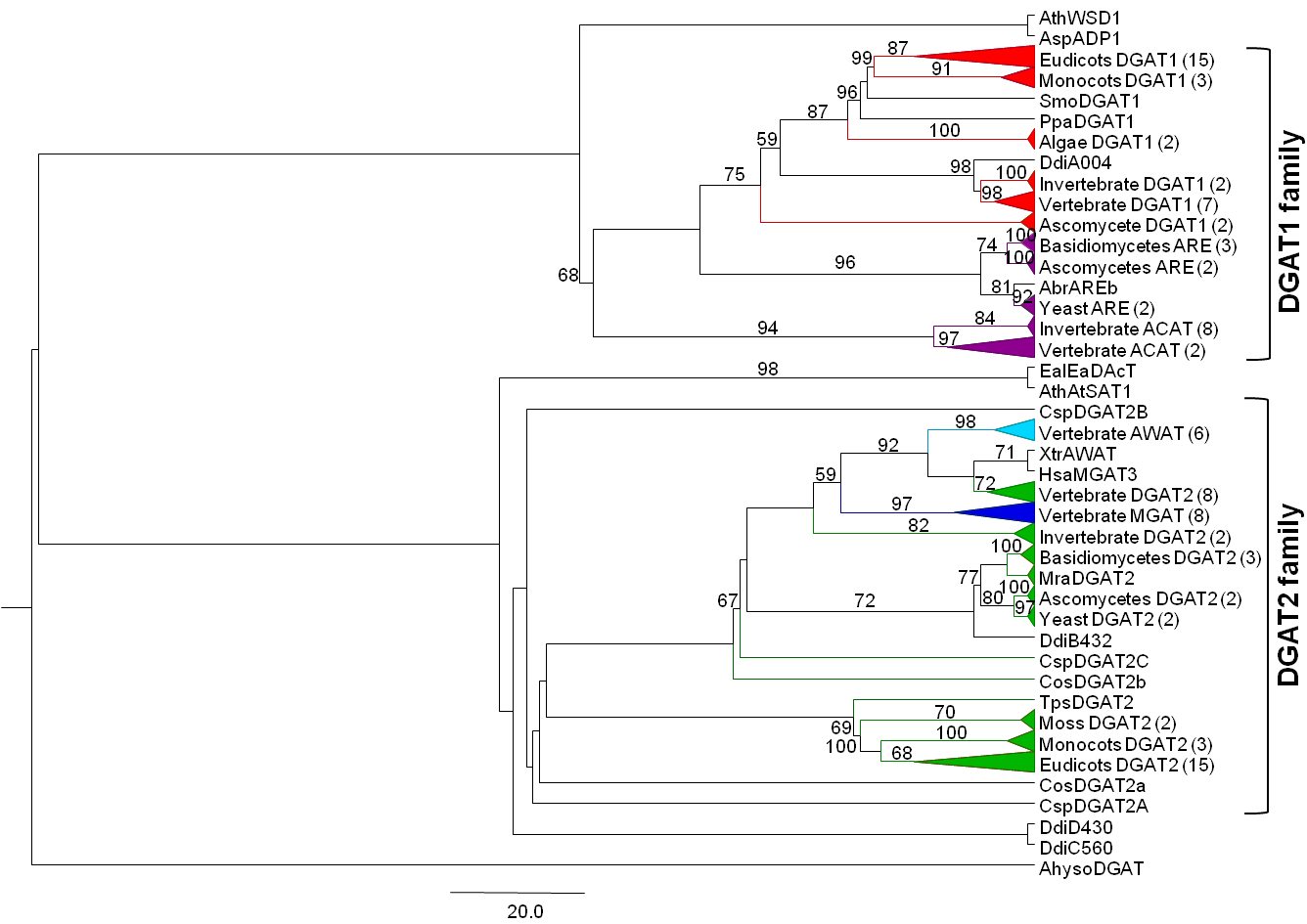

Supplement: Additional file 3 — Phylogenetic tree of the DGAT1 and DGAT2 gene families reconstructed by the Maximum likelihood (ML) method. A total of 151 protein sequences from eukaryotic species and 344 sites were included in the analyses. The bootstraps values are labeled above the branches. Only values > 50% are presented. Numbers within brackets correspond to the number of species within each group. The enzymes are represented by different colors in the phylogenetic tree: DGAT1 (red), DGAT2 (green), ACATs (purple), MGAT (dark blue) and AWAT (light blue). Taxa terminologies are abbreviated using the first letter of the genus and two letters of the species name: Alternaria brassicicola (Abr), Arabidopsis thaliana (Ath), Dictyostelium discoideum (Ddi), Euonymus alatus (Eal), Homo sapiens (Hsa), Mortierella ramanniana (Mra), Thalassiosira pseudonana (Tps), Xenopus tropicalis (Xtr). [file 1471-2148-11-263-S3.TIFF]

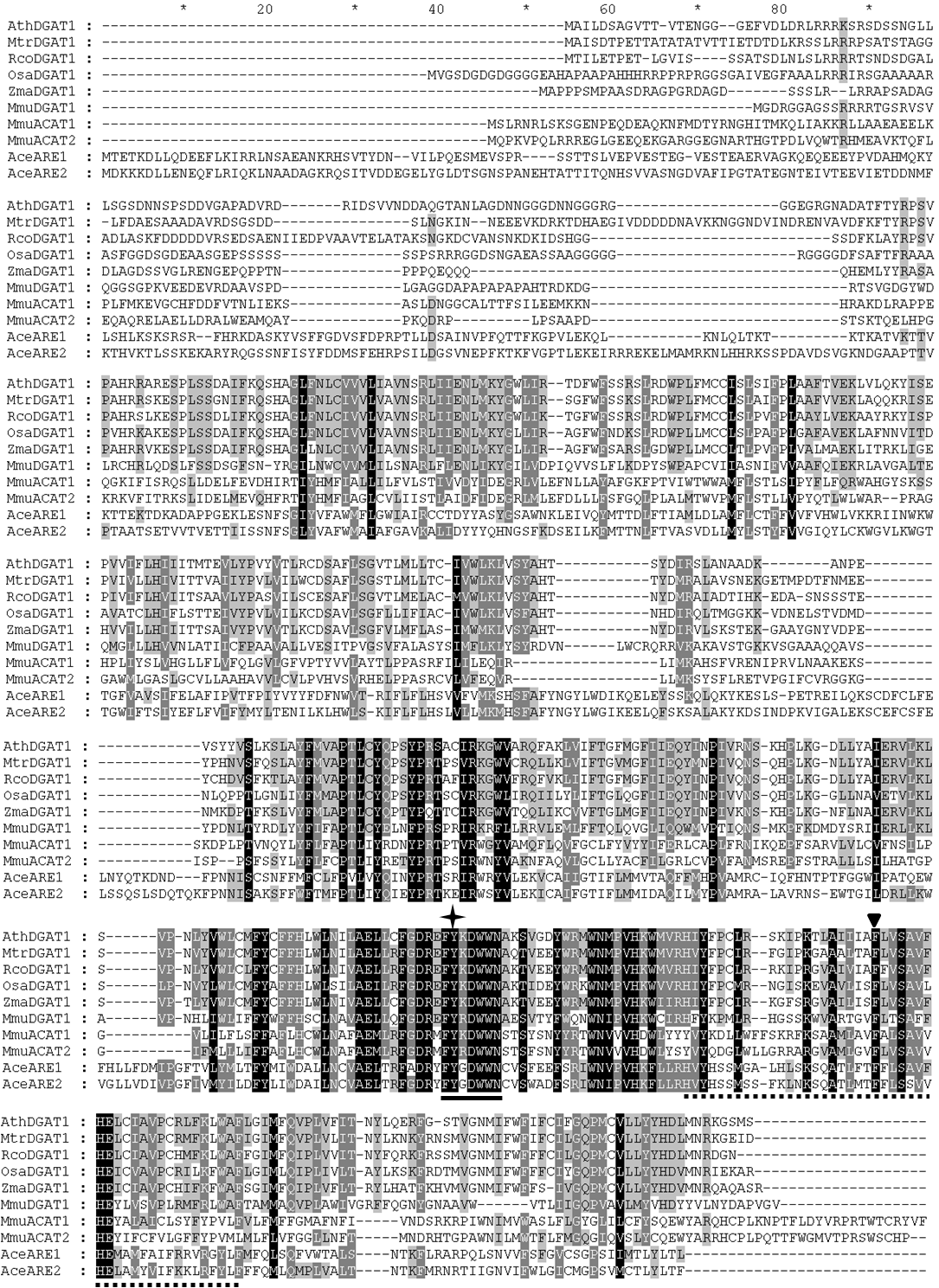

Supplement: Additional file 4 — Multiple sequence alignment of predicted amino acid sequences of DGAT1 proteins. DGAT1 sequences from A. thaliana (Ath), M. truncatula (Mtr), R. comunis (Rco), O. sativa (Osa), Z. mays (Zma) and M. musculus (Mmu), and ACAT proteins from M. musculus and S. cerevisieae (Sce) were aligned. Identical residues are shaded black, and similar residues are shaded gray. The DAG/phorbol ester binding signature motif is underlined with a dotted line; the triangle (▼) shows the conserved phenylalanine. The conserved motif between DGAT1 and ACATs is underlined. [file 1471-2148-11-263-S4.TIFF]

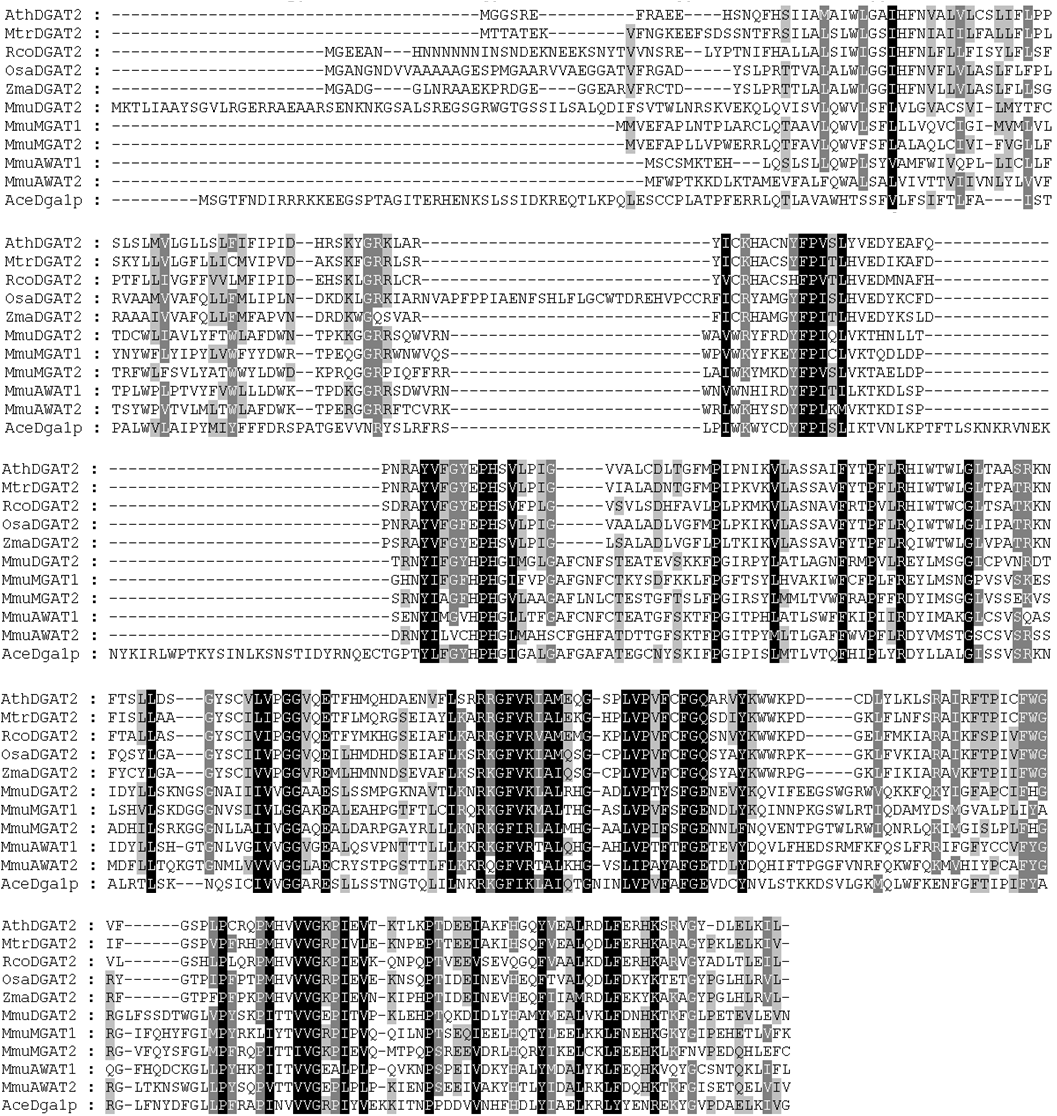

Supplement: Additional file 5 — Multiple sequence alignment of deduced amino acid sequences of DGAT2 proteins. DGAT2 sequences from A. thaliana (Ath), M. truncatula (Mtr), R. comunis (Rco), O. sativa (Osa), Z. mays (Zma), M. musculus (Mmu) and S. cerevisieae (Sce) were aligned with MGAT and AWAT proteins from M. musculus. Identical residues are shaded in black, and similar residues are shaded in gray. [file 1471-2148-11-263-S5.TIFF]

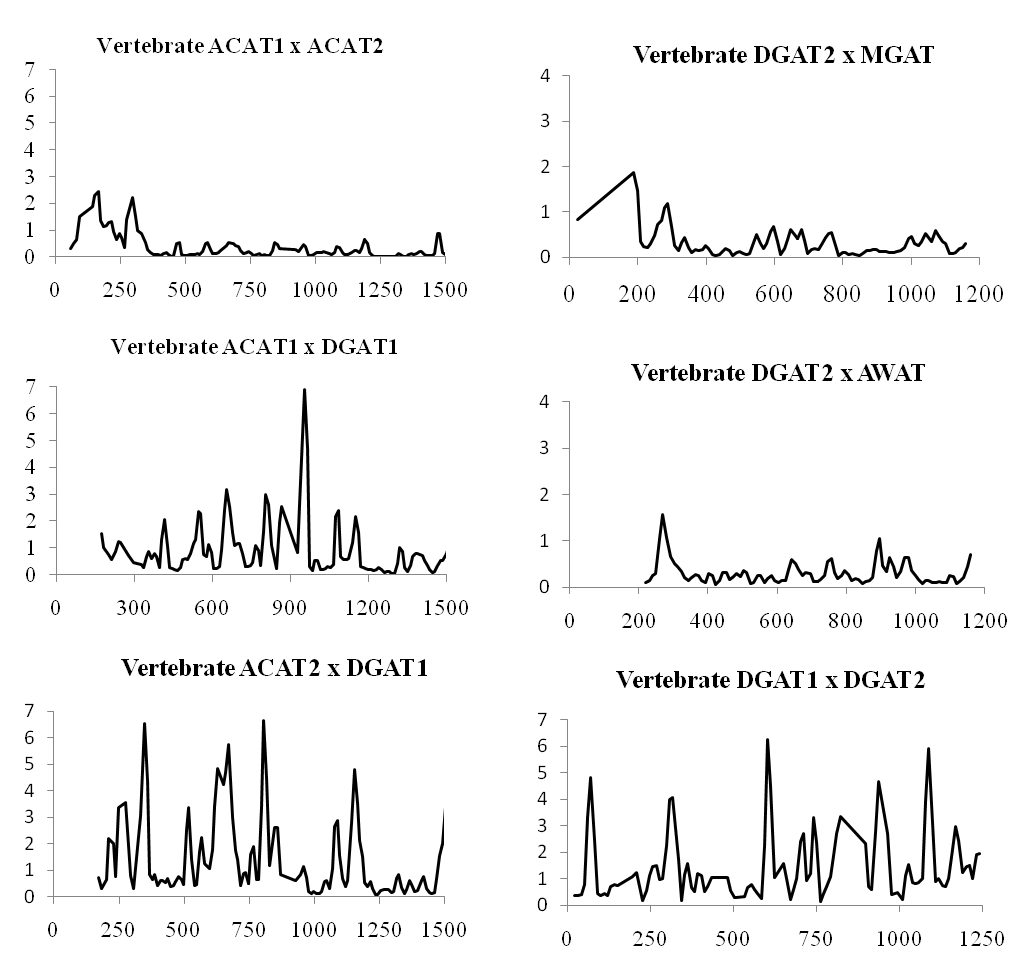

Supplement: Additional file 6 — Estimation of Ka/Ks rates on vertebrate sequences. Substitution rates between full-length cDNA sequences of vertebrate DGAT1, DGAT2, ACAT1, ACAT2, MGAT and AWAT. Comparisons were performed between ACATs and DGAT1, MGATs and DGAT2 and between AWAT and DGAT2. [file 1471-2148-11-263-S6.TIFF]

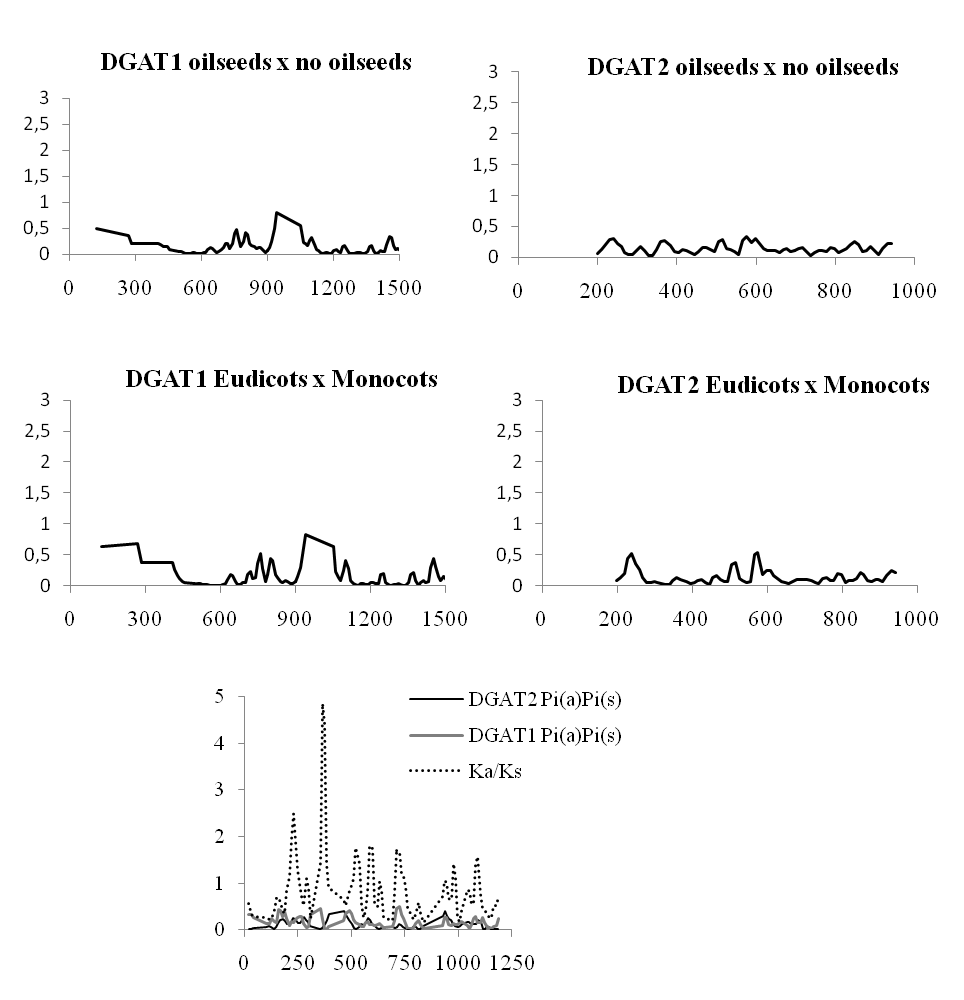

Supplement: Additional file 7 — Estimation of Ka/Ks rates on plant sequences. Substitution rates between full-length cDNA sequences of plant DGAT1 and DGAT2. Comparisons were performed for DGAT1 and DGAT2 between monocotyledon and eudicotyledon, between oilseed and non-oilseed plants and between DGAT1 and DGAT2 from all plant species. [file 1471-2148-11-263-S7.TIFF]
